# Genome-Wide Analysis of Protein Disorder in *Arabidopsis thaliana*: Implications for Plant Environmental Adaptation

**DOI:** 10.1371/journal.pone.0055524

**Published:** 2013-02-07

**Authors:** Natalia Pietrosemoli, Juan A. García-Martín, Roberto Solano, Florencio Pazos

**Affiliations:** 1 Computational System Biology Group, National Centre for Biotechnology (CNB-CSIC), Madrid, Spain; 2 Plant Molecular Genetics Department, National Centre for Biotechnology (CNB-CSIC), Madrid, Spain; 3 Department of Bioengineering, Rice University, Houston, Texas, United States of America; University of Toronto, Canada

## Abstract

Intrinsically Disordered Proteins/Regions (IDPs/IDRs) are currently recognized as a widespread phenomenon having key cellular functions. Still, many aspects of the function of these proteins need to be unveiled. IDPs conformational flexibility allows them to recognize and interact with multiple partners, and confers them larger interaction surfaces that may increase interaction speed. For this reason, molecular interactions mediated by IDPs/IDRs are particularly abundant in certain types of protein interactions, such as those of signaling and cell cycle control. We present the first large-scale study of IDPs in *Arabidopsis thaliana*, the most widely used model organism in plant biology, in order to get insight into the biological roles of these proteins in plants. The work includes a comparative analysis with the human proteome to highlight the differential use of disorder in both species. Results show that while human proteins are in general more disordered, certain functional classes, mainly related to environmental response, are significantly more enriched in disorder in Arabidopsis. We propose that because plants cannot escape from environmental conditions as animals do, they use disorder as a simple and fast mechanism, independent of transcriptional control, for introducing versatility in the interaction networks underlying these biological processes so that they can quickly adapt and respond to challenging environmental conditions.

## Introduction

To some extent contradicting the classical protein structure-function relationship paradigm, there is a type of proteins whose function is indeed determined by their intrinsic lack of a fixed 3D structure. These proteins, known as “intrinsically unstructured (or disordered) proteins” (IUPs/IDPs) are totally or partially unfolded in their native state [1], [2], [3], [4]. First regarded as having little biological interest, these proteins (or protein regions -IDRs-) were considered as “junk proteome” not performing important functions within the cell. Research on these polypeptides was delayed not only by those misconceptions but also by the fact that structural and molecular biology techniques were designed to work under the paradigm of the structure-function relationship. Nevertheless, from being considered as rare “trash” proteins without important functions, IDPs are finally being recognized as, i) a widespread phenomenon, and ii) proteins with very important functions within the cell.

IDPs and IDRs were first detected as missing segments in protein structures determined by X-ray diffraction or as those for which not enough constraints existed for defining a fixed structure in NMR spectra, both indicative of highly mobile regions. At the sequence level, IDPs and IDRs are characterized by long stretches of polar and charged residues, which impede the formation of hydrophobic cores and the subsequent folding [2], [3], [5], [6]. Due to this particular amino acid composition, these regions without defined structure were associated to “low complexity” or “composition biased” sequence segments [7], [8], [9], [10]. Computational methods for detecting low complexity regions existed long ago before IDPs were found, and consequently they were among the first tools for predicting IDRs in proteins. Later, as the number of experimentally detected IDPs increased, it was possible to develop specific methods trained with them [10], [11], [12]. These methods based on machine learning, together with later ones based on physical properties [13], [14] were used for scanning large collections of protein primary sequences in the search for IDRs. This prediction of IDPs and IDRs in complete proteomes rendered a surprising result: a large fraction of these proteomes was predicted to be unstructured, especially in eukaryotic organisms. In fact, between 5%–15% of the proteins are expected to be fully disordered, and about half of the proteins to have at least one long disordered region of 30 residues or more [12], [15].

In parallel to their abundance estimation, it started to become evident that these IDPs play central roles within the cell [16], [17]. They are involved, among other, in the control of the cell cycle, transcriptional regulation, signaling cascades, and chaperone activity [6], [18], [19], [20]. Within these biological processes, IDRs have two main molecular functions, arisen from their lack of fixed structure: flexible connectors (“springs”, “linkers”, etc.) between globular domains, and molecular recognition sites. The molecular interaction mediated by IDRs may be either permanent or transient and, in some cases, these unstructured proteins (or segments) acquire structure upon binding to a partner [5]. The interactions mediated by IDPs/IDRs have characteristics of affinity/specificity that are very suitable for certain types of protein interactions, such as those occurring in the previously mentioned biological processes (e.g. signaling cascades, cell cycle regulation). Given their intrinsic flexibility and ability to accommodate to different binding surfaces, IDPs are able to recognize and interact with multiple partners, thus relating protein disorder with interaction versatility [5], [6], [21]. Similarly, this same conformational flexibility confers them a larger interaction surface that may translate into an increased speed of interaction [22].

Due to the importance and widespread occurrence of these proteins and their special structural and functional roles, particularly in terms of molecular interactions, the repertoires of IDPs/IDRs of a given organism can provide important information of its biology. Indeed, IDPs content has been previously proposed to be related to organismal complexity and its capacity to adapt to different environments [12], [23], [24], [25]. Thus, long (>30 residue) disordered segments are found to occur in 2.0% of archaean, 4.2% of eubacterial and 33.0% of eukaryotic proteins [12]. Similarly, proteomes of organisms adapted to low temperatures or high salt conditions are more disordered than those from thermophiles or non-adapted bacteria [26], [27].

Specifically in the case of plants, being sessile organisms, they are unable to escape from environmental challenges as animals can do. As a consequence, phenotypic plasticity (the capacity to adapt their phenotype to changing conditions) is particularly important in plants to adapt to and survive in changing environments. Phenotypic plasticity requires the integration of external information with the basal genetic/developmental programs, and is achieved through complex signaling networks in plants [28].

Bioinformatic and experimental analyses of a limited number of proteins from plants have found disordered regions in proteins involved in signaling and environmental adaptation, which, similar to the trend in bacteria described above, could suggest a relationship between protein disorder and phenotypic plasticity [29], [30], [31]. However, a whole-proteome analysis of the functional role of protein disorder in plants that could support this hypothesis is still missing.

In this work, we performed the first large-scale functional study of the repertoire of IDPs/IDRs in *Arabidopsis thaliana,* the most widely used model organism in plant biology. The study includes a systematic comparison of the role of protein disorder in *A. thaliana* and *H. sapiens*. While the intrinsic disorder characteristics of the human genome have been vastly characterized [32], [33], [34], their role in Arabidopsis is just starting to be unveiled and remains confined to specific biological functions [31], [35], [36]. Our large-scale comparative analysis of protein disorder in these two organisms provides insights on the specific functional roles that this phenomenon plays in *A. thaliana*. In particular, IDPs/IDRs in *A. thaliana* seem to be specifically related to certain functions within this organism, such as hormonal and non-hormonal signaling, response to external stimuli and post-translational protein modifications (chaperon activity), processes which underlie phenotypic plasticity and adaptation to environmental stress. Our results point to the hypothesis that plants might be using disordered proteins as a simple mechanism, independent of transcriptional control, for introducing versatility in the interaction networks underlying certain biological processes in order to adapt and respond to changing environmental conditions.

## Results

### Overall Disorder Content

**Figure 1:**
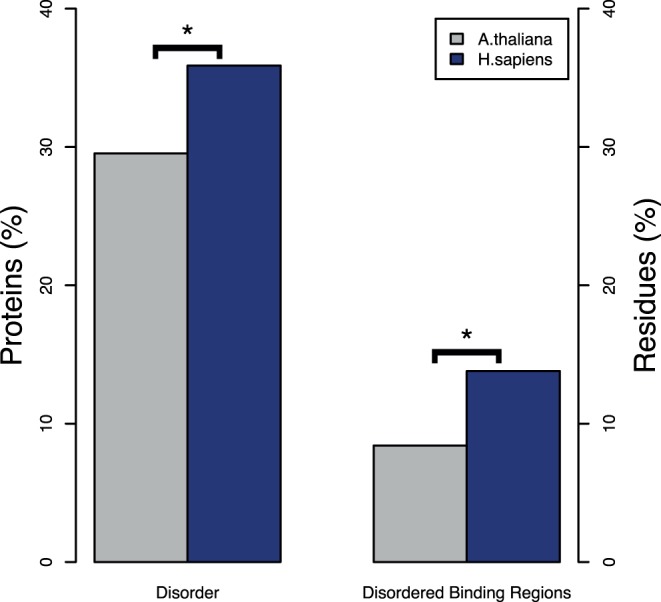
Overall predicted global disorder and disordered binding regions in *A. thaliana* and *H. sapiens* proteins. Left: percentages of disordered proteins (disordered proteins criterion: those proteins containing at least 50% disordered residues based on Disopred predictions). Right: average percentages of disordered residues involved in binding (DBRs), as predicted by ANCHOR. The stars denote significant differences evaluated with the same Chi-square tests described in the Methods section and illustrated in Figure 1 but using all proteins (i.e. not restricted to a particular GO functional class).

The proteome of Arabidopsis was, on average, less disordered than that of human. Table 1 shows a number of figures representing different quantifications of “disorder” in *A. thaliana* and *H. sapiens*. There were significantly more disordered proteins in human (defining “disordered protein” as one with > = 50% of disordered residues) with respect to *A. thaliana*: 35.9% vs. 29.5% (Figure 1; Chi-square test; p-value: <2.2E–16). The percentage of proteins with at least one “long disordered region” (LDW) was also higher in human (68.5% vs. 57.2%, Chi-square; p-value: <2.2E–16;) and so was the average number of LDWs per protein (1.46 vs 0.96; Wilcoxon raked sum test; p-value <2.2E–16). Furthermore, the average number of residues that fell into these LDWs was also higher in human (27.0 vs 19.7; Wilcoxon raked sum test; p-value <2.2E–16). Although these results are based on DISOPRED predictions, the tendency was also maintained for the other predictors (see Additional Data File S1, Table 1S) and so was its statistical significance.

**Table 1:** Summary of intrinsic disorder metrics for *A. thaliana and H. sapiens*.

	*A. thaliana*	*H. sapiens*
**Mean content of disorder**	29.5%	35.9%
**Proteins with at least one LDWs**	57.2%	68.5%
**Mean number of LDWs**	0.96	1.46
**Mean number of residues belonging to LDW**	19.67%	27.04%
**Proteins with at least one DBR**	50.7%	66.3%
**Mean DBR per protein**	2.34	5.11
**Mean resides belonging to DBR**	8.4%	13.8%

Results shown for Disopred (disorder prediction) and ANCHOR (Disorder binding regions, DBRs). For results with other predictors see Additional Data File S1, Table 1S.

**Figure 2:**
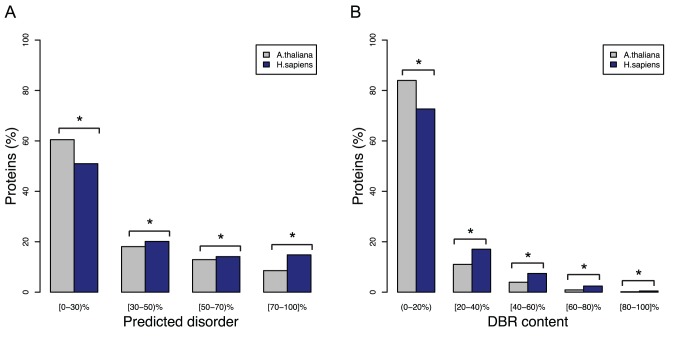
Fraction of proteins with different degrees of predicted disorder and disordered binding regions in *A. thaliana* and *H. sapiens*. While the left panel of Figure 1 shows the content of highly disordered proteins (>50%), this is intended to evaluate this for different degrees of disorder. A) Protein disorder (as the percentage of disordered residues with respect to the sequence length) is binned into different ranges. Data based on Disopred predictions. B) The same for disordered residues involved in binding, as predicted by ANCHOR. The significance of the differences is evaluated as in Figure 1.

**Figure 3:**
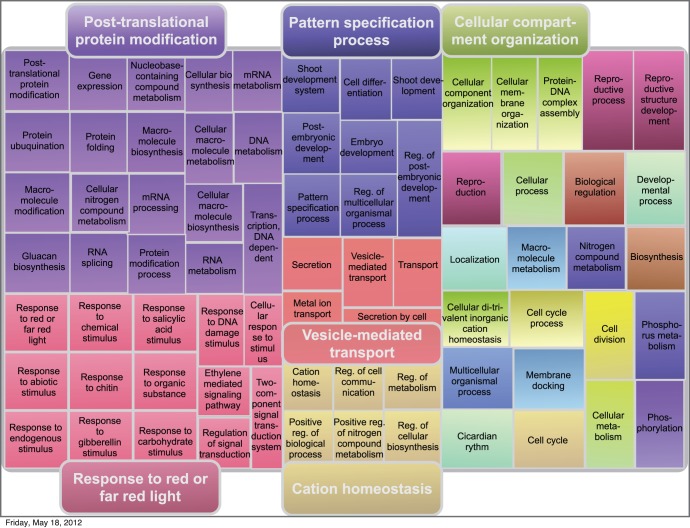
Representation of the GO “Biological Processes” significantly enriched in disordered proteins in *A. thaliana*. Disordered proteins here correspond to those with one or more “long disordered windows” (LDW) based on Disopred predictions. Figure adapted from REVIGO, a system for summarizing and visualizing lists of GO terms. Each rectangle represents a cluster of related terms labeled according to a representative term. Rectangles are grouped in “superclusters” (identified with the same color) based on SimRel semantic similarity measure.

In order to assess whether this difference was only observed for highly disordered proteins (> = 50%) or it was also evident for other ranges of sequence disorder, proteins were grouped according to the percentage of predicted disorder of their sequence (Figure 2A). The distribution was shifted to lower percentages of disorder (0–30%) in Arabidopsis, while in human it was shifted to higher disorder content (30–100%). Again, these differences were statistically supported and predictor-independent (see Additional Data File S1, Figs. 1S, 2S, 3S), with the exception of the 30–50% bin for the VSL2 predictor, for which there was not statistical difference between both organisms.

Similarly, human proteome was also more enriched in predicted disordered regions potentially involved in protein-protein interactions (Disorder Binding Regions, DBRs) (Figure 1 and Table 1). While 50.7% of Arabidopsis proteins had at least one DBR, the proportion for human was of 66.3% (Chi-square; p-value <2.2E–16). The average number of DBRs per protein was also higher in human (5.11 vs 2.34, Wilcoxon raked sum test; p-value <2.2E–16). In the same manner, the average content of disordered-binding residues was higher in human than in Arabidopsis: 13.8% vs. 8.4% (Wilcoxon rank sum test; p-value <2.2E–16) (Figure 1). When proteins were grouped according to intervals of DBR residues content, there was always statistical difference between the number of DBR residues for both species, with more disordered binding residues in *H. sapiens* (Figure 2B).

### Disorder and Functional Categories

In the first part of this section we evaluated which functional categories were significantly enriched in disordered proteins in *A. thaliana*. In the second part, we performed a comparative analysis to detect functional classes that were distinctively associated to disorder in this organism with respect to human. Functional classes found in both evaluations would correspond then to those being significantly enriched in disorder in Arabidopsis while being also more disordered in this organism respect to human. Similarly, a GO term appearing in the first evaluation but not in the second one would represent a functional class significantly disordered in Arabidopsis but with a similar level of disorder in human. Finally, a GO term showing up in the second test but not in the first one would correspond to a function that, while not being specially enriched in disordered in Arabidopsis, it was still much more disordered than in human. The complete sets of GO terms found in the two evaluations are shown in Additional Data File S2 (Arabidopsis) and Additional Data File S3 (Arabidopsis vs. human). To facilitate the biological interpretation of these large sets of GO terms, we analyzed the lists with REVIGO, which summarizes long lists of GO terms into a slimmer list of statistically significant terms (see Methods).

#### Functional categories significantly disordered in *A. thaliana*

Figure 3 shows the REVIGO representation summarizing GO biological processes that were detected by DAVID as overrepresented (p-value < = 0.05) in the set of disordered proteins of Arabidopsis (those with at least one LDW according to DISOPRED predictions; see Methods). The complete list of 145 terms is available in the Additional Data File S2.

Functional categories enriched in disordered proteins in Arabidopsis (Figure 3) included “post-translational protein modification” (comprising nucleic acid metabolism, gene expression, protein synthesis and maturation) and a category labeled by REVIGO as “response to red or far red light”. The latter, in addition to light signaling, included “response to endogenous and abiotic stimulus” and most of the hormonal signaling pathways. Therefore, in spite of the GO term chosen by REVIGO to label it, this category could be better summarized as “response to stimulus”. Other significantly enriched terms were ”pattern specification”, “transport”/”secretion”, “cation homeostasis”, “cellular compartment organization” (mostly referring to chromatin and nucleosome assembly), “cell cycle”, and “reproduction”. Very similar results were obtained with other disorder predictors and other disorder criteria (See Additional Data File S1). In summary, these functional classes could be summarized as “signaling”, “development”, “cell cycle” and “response to stress” (light, abiotic, etc.), and they were represented, among others by proteins belonging to hormonal signaling pathways or transcription factors.

#### Functional categories more disordered in *A. thaliana* than in *H. sapiens*

**Figure 4:**
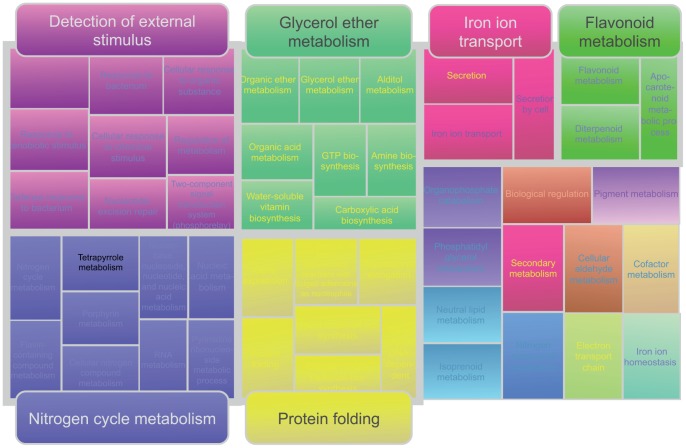
Representation of the GO “Biological Processes” comparatively enriched in disordered proteins in *A. thaliana* respect to *H. sapiens.* Disordered proteins here are again those with 1 or more LDWs based on Disopred predictions. Same REVIGO representation adaptation as in Figure 3.

Figure 4 shows the REVIGO representation summarizing the GO biological processes with a significantly higher proportion of disordered proteins in Arabidopsis as compared to human (p-value < = 0.05). As in the previous section, disordered proteins corresponded to those with at least one LDW according to DISOPRED predictions (see Methods). The complete list of terms is available in the Additional Data File S3. While 145 GO terms were significantly enriched in disorder in Arabidopsis (previous section), there were only 88 terms for which the disorder degree was significantly higher than in human.

**Figure 5:**
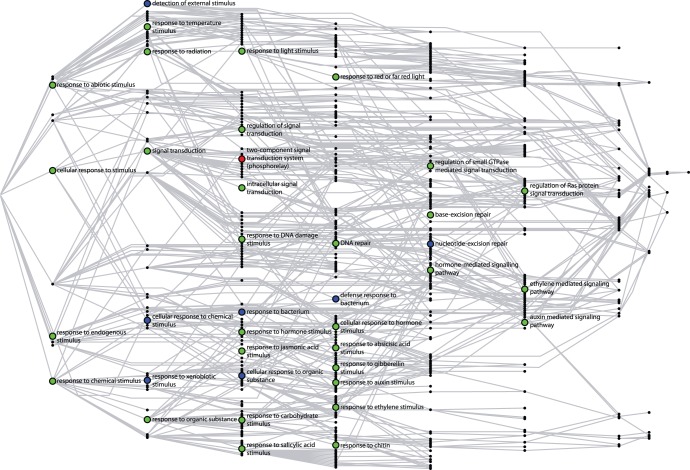
Subgraph of biological process “Response of stimulus” (GO:0050896). Green nodes correspond to those GO:BP terms significantly enriched in disorder in Arabidopsis. Blue nodes correspond to those GO terms enriched in disorder in Arabidopsis compared to human. The red node represents the only common term between these two sets.

Again, we found enrichment in categories associated to “detection and response to stimulus”. In this case, however, most of such categories were related to external stimulus (Figure 4). Figure 5 shows a detailed view of the “response to stimulus” GO:BP subgraph, highlighting the terms which are enriched in disorder in Arabidopsis (previous point), as well as those more enriched in Arabidopsis when compared to human. It can be seen that the latter terms were more related to external stimulus.

**Figure 6:**
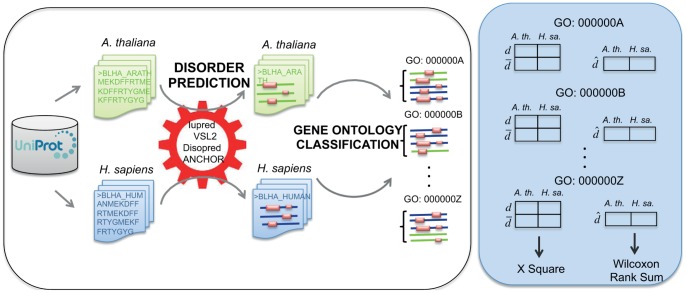
Schematic representation of the methodology used for the comparative study of protein disorder in *A. thaliana* and *H. sapiens*. For each organism (Arabidopsis (green) and human (blue)) protein sequences and their corresponding Gene Ontology annotations are retrieved from Uniprot. For each protein, disordered regions (pink) are calculated using 3 different methods (Iupred, VSL2 and Disopred), and disordered-binding regions (DBRs) are predicted using ANCHOR. Proteins are assigned to GO:BP functional classes. For each GO functional class, a comparative analysis of the disorder levels of the proteins of each organism is performed, using different criteria for quantifying disorder in that given GO class. For those disorder criteria that assign a “yes/no” label to a given protein, contingency tables are constructed with the counts of disordered and not-disordered proteins in both organisms and a Chi-squared test is applied to them. For those criteria that quantify the disorder of a given protein, the tables contain the average values of that figure for both organisms, and a Wilcoxon rank sum test is applied.

The fact that the terms “response to endogenous stimulus”, “cell cycle”, etc. were no longer enriched indicates that proteins of these particular categories had similar disorder content in human and Arabidopsis. In contrast, “Protein folding” (including nucleic acid metabolism, gene expression, protein synthesis and maturation) was again present, indicating that these processes were more disordered in Arabidopsis than in human. Other functional categories with significant disorder included those related to nitrogen metabolism and other molecules (flavonoids, glycerol, isoprenoids, cofactors, pigments). Using other disorder predictors and disorder criteria provided similar results (See Additional Data File S1, Figs. 4S, 5S, 6S), especially for those processes related to the response to external stimulus, which repeatedly appeared as more disordered in Arabidopsis than in human, independently of the predictor and criteria used.

In summary, GO functional classes that were more disordered in Arabidopsis compared to human can be divided into two major related functions: “detection and signaling of external stimulus” (including chaperone activity induced by stress, related to “protein folding”) and “secondary metabolism”. In the case of plants, the latter is intrinsically related to the response to external stimulus, because plants have developed secondary metabolites as major tools to cope with environmental stress.

Among proteins annotated under “detection of external stimulus” and “nucleotide-excision repair”, it was remarkable the high amount of those involved in perception and signaling of light quality, which is the most influential external stimulus in plant development.

To discard that the differences in disorder observed for these GO terms are due to biases or peculiarities of the GO annotations of these two model organisms, we took the subsets of orthologous proteins within these classes and calculated their average disorder in Human and Arabidopsis (using four different disorder metrics). The results are shown in Additional Data File S4. 80 out of the 88 classes present some degree of orthology. For all these 80 classes the Arabidopsis orthologs are more disordered than their Human counterparts (except one, when evaluated with two of the four disorder metrics). Both, the difference in disorder and the bias in favor of Arabidopsis of this difference are statistically supported (see Additional Data File S4). For example, the average percentage of disordered residues for the orthologs within these 80 classes is 27.2% for A. thaliana and 19.9% for Human (P-value of one-tailed t-test: 2.6E–4).

Finally, we conducted the same comparative analysis of Arabidopsis vs. human for disordered binding regions (DBRs). In this case, the functional categories we found are also related to “detection/response to external stimulus”, “defense against bacteria”, “multi-cellular processes”, and number of metabolic processes (See Additional Data File S1, Fig. 7S).

## Discussion

The success of evolution in generating organism complexity has been paralleled by the increase in complexity in the underlying biological processes. One can conceive two main ways for increasing the plasticity and complexity of a biological process supported by a network of protein-protein interactions: either increasing the number of proteins or increasing the number of interactions (“wiring”). Protein-protein interactions mediated by unstructured regions are recognized as a way of conferring plasticity to protein interaction networks [5], [6], [21]. Additionally, due to the physico-chemical characteristics of the interactions mediated by flexible/disordered regions, they are frequently involved in transient interactions with multiple partners [37]. Accordingly, increasing the disorder content of a particular subnetwork (biological process) of the interactome is a way to increase its “wiring”, the possible connections between the proteins and, consequently, the plasticity of the system, all without increasing the number of proteins involved. In fact, disorder content has been proposed to positively correlate with what one intuitively recognizes as “organism complexity” [12], [25], [38]. This could be explained by the fact that higher organisms use disorder as a means (among others) to obtain more complex and “sophisticated” interactomes. Our results show that the human proteome is, globally, more disordered than the one of Arabidopsis. This trend is supported regardless of the predictor and criterion used for defining disorder. At the same time, both human and Arabidopsis, as complex eukaryotes, are also much more disordered than bacteria.

In concordance with previous observations [6], [18], [19], [20], we found that disorder in Arabidopsis is involved in biological processes rich in transient interactions with multiple partners (e.g. cell cycle, signaling, DNA and RNA metabolism –including splicing–). These processes, which are generally more complex in eukaryotes than in prokaryotes or that may even represent new acquisitions of evolution (e.g. splicing), are the prototypical processes that have been previously related to disorder in higher organisms. At the protein level, the characteristic example is cancer-related p53, a key protein for cell cycle control, which is disordered in almost half of its length [37].

Taken together, our results on the overall disorder content in Arabidopsis and on its disorder enriched processes are consistent with the previously hypothesized correlation between organism/process complexity and protein disorder.

It is striking, however, that despite the fact that all intrinsic disorder criteria evaluated in this study point to higher disorder levels for human, we find some functional classes for which disorder is significantly higher in Arabidopsi*s.* This difference in disorder is also evident when considering only the “comparable” (orthologous) proteins between both organisms, discarding that it could be an artifact due to biases in the GO annotations of these two genomes. It has been shown that such biases can cause problems for some studies involving GO annotations of different organisms if certain cautions are not taken [39].

These GO classes are related to processes such as environmental perception and response –for which plants have developed more complexity– and are fundamental for their adaptation. The ability to accommodate its phenotype to changing environmental conditions, or *phenotypic plasticity*, is very important for adaptation and survival of any organism. In the case of plants, plasticity is particularly relevant since these sessile organisms cannot escape from environmental challenges as animals can do [28]. Specifically, plant plasticity depends on the capacity to identify the challenge, integrate the external information through signaling pathways, and finally change the basal developmental programs to stress programs (which include the production of secondary metabolites) to adapt and survive to those threats.

There are processes for which plants have developed particularly complex mechanisms. Light, for instance, is probably the most influential external clue for plant development, and plants have developed complex perception (photoreceptors) and signal transduction mechanisms to finely tune their growth and development according to light quality and intensity [40], [41], [42], [43]. Indeed, a recent study highlights the importance of intrinsic disorder in plant chloroplasts [36]. Remarkably, in our results terms related to “response to light stimulus” appear as disordered in Arabidopsis and also in the comparison to human. Other GO term enriched in disordered proteins in Arabidopsis as compared to human is “nucleotide-excision repair”, which includes (among others) proteins involved in UV light perception and response. Thus, processes for which plants have developed mechanisms more complex than humans appear more disordered, further supporting the hypothesized correlation between complexity and disorder.

Another example of complexity in plant development/function is their ability to adapt to abiotic stress, such as drought, salinity or the cell desiccation that occurs during seed development. Consistent with this complexity, several GO terms enriched in disordered proteins (compared to human) are related to protein folding or abiotic-stress related signaling. Moreover, among the plant proteins for which disorder has been previously studied in detail, ERD10 and ERD14 are examples of chaperones whose structural disorder provides the flexibility to interact with many different partners and prevent their denaturation and aggregation [30].

An additional set of GO terms significantly disordered in Arabidopsis as compared to human is related to secondary metabolism (“flavonoid”, “isoprenoid”, “pigment”, “nitrogen”, “vitamin”, “cofactor”, etc.), which, in many cases, are evolutionary acquisitions of plants to cope with environmental stress and adaptation. Some flavonoids and anthocyanins, for instance, are produced by plants to protect from UV radiation (another GO term more disordered in Arabidopsis, as pointed above), whereas other secondary metabolites are involved in attracting pollinators or defending from predators [44], [45], [46]. In the case of nitrogen, it is often a limiting factor for plant growth. Multiple nitrogenous compounds are involved in different functions in plants, including storage of nitrogen, but they are also related to defense and signaling [46].

Our systematic comparative study shows that while human proteome is globally more disordered than that of Arabidopsis, there are some particular GO functional classes that are more enriched in disordered proteins in Arabidopsis when compared to human. Interestingly, these functional classes are related to processes for which plants have developed particularly complex mechanisms, such as adaptation to the environment. The general relationship between disorder and processes related to the response to environmental stimuli had been previously discussed [25] and our results add support to that.

It has been already proposed that “increasingly integrating protein disorder into the toolbox of a living cell was a crucial step in the evolution from simple bacteria to complex eukaryotes” [24]. Our results add support to this hypothesized correlation between organism/process complexity and protein disorder, and suggest that plants have used disorder as an evolutionary tool to increase complexity in their biological/protein networks. This increased complexity is particularly evident in those networks underlying phenotypic plasticity and adaptation to environmental stress.

## Materials and Methods

An overview of the methodology is shown in the Figure 6.

### Sequence Datasets

The dataset for the analysis was constructed taking the proteome sequences of *A. thaliana* and *H. sapiens* from the Protein Knowledgebase (UniProtKB, release 2011 04) [47]. We used the search engine of this resource to look for “A. thaliana” and “H. sapiens”, and selected the “complete proteome” option, resulting in two sets of 32.764 and 35.346 sequences including both canonical proteins and isoforms. This dataset was filtered out for repeated, fragmented and proteins containing non-standard residues (such as Selenocysteine) and ambiguous residues (e.g. B, X, Z), that may not be tractable by certain disorder prediction algorithms.

This final dataset contains 32.398 proteins for *A. thaliana* (coming from 31.304 genes) and 35.244 proteins for *H. sapiens* (from 20.154 genes).

### Functional Annotations

In order to associate functional terms to the protein sequences described above, we use the functional vocabulary defined by the Gene Ontology Consortium (release 2011 04) [48]. These Gene Ontology (GO) terms describe different functional aspects of gene products and are divided into three independent categories (subontologies): “biological process”, “cellular component” and “molecular function”. The GO annotations for our sequences were also retrieved from UniprotKB. Terms that were labeled by the GO Consortium as “obsolete” were not included in the analysis. Arabidopsis genes were annotated with a total of 4.278 GO functional terms from the three subontologies and human genes were annotated with a total of 8.836 GO terms.

The Gene Ontology is structured as a directed acyclic graph where the terms are related by parenthood relationships, in such a way that it can be navigated from very general (e.g. “enzyme”) to more specific functions (e.g. “coenzyme F390-G hydrolase activity”). In general, the original GO annotations contain only the most specific terms that can be assigned to a given protein. We expanded the original set of GO terms associated to a given protein (see above) by including all the ancestors of these GO terms. In this way, we ensured that any pair of proteins can be functionally compared at the GO level at which they have the common ancestor. For example, two proteins annotated respectively with the two GO terms mentioned above would be compared as “enzymes”. This expansion resulted in 6.410 GO terms for *A. thaliana* and 12.690 GO terms for *H. sapiens*. 4.380 of these terms were used to annotate both *A. thaliana* and *H. sapiens* proteins, and hence those are the ones used for the comparative analysis.

### Protein Disorder Prediction

The prediction of intrinsic protein disorder was carried out using three different tools: Disopred v2.4 [49], VSL2 [50] and Iupred [14]. The first two disorder predictors are based on linear support vector machines. The later is based on the pairwise energy content estimated from residue composition. All three of them take a single protein sequence as input and provide as output a disorder probability in the 0.0–1.0 range for each residue. For converting these values into a binary (“ordered” vs. “disordered”) prediction at the residue level we used for all the predictors their default thresholds (0.5 for VSL2, and Iupred and 0.05 for Disopred).

For every protein in the two datasets (*A. thaliana* and *H. sapiens*), we defined two disorder metrics: a) relative disorder content (i.e. percentage of disordered residues in whole protein), and b) number of long disordered windows, LDW (i.e. number of protein regions with at least 30 consecutive disordered residues). These two metrics represent two different disorder criteria and have been previously used in the literature.

Additionally, we extract the disordered regions predicted to be involved in protein-protein interactions, given the important role protein disorder plays in binding. We used the ANCHOR tool [51], which is based on the IUPred program mentioned above. This method takes an amino acid sequence as an input and predicts binding regions that are disordered in isolation and may go under a disorder-to-order transition upon binding. The output of this method is the same as those of the methods described above and hence it was transformed in the same manner.

### Evaluating the Disorder in Gene Ontology Functional Classes

We want to evaluate i) the GO classes enriched in disorder in Arabidopsis, and ii) those differentially enriched in Arabidopsis respect to Human.

To evaluate whether a given GO class was significantly associated to disordered proteins in Arabidopsis (taking into account the average disorder of all classes) we conducted an “enrichment analysis” test [52] as implemented in the DAVID tool [53]. We used the number of proteins with at least one “long disordered window” (LDW) based on DISOPRED predictions as the quantification of the disorder of a given GO class, and the following parameters as input for DAVID: Background: “Arabidopsis thaliana”. Gene Ontology subontologies “GOTERM BP ALL”, “GOTERM MF ALL”, and “GOTERM CC ALL”. A “Functional Annotation Chart” was generated listing all the annotation terms and their associated genes. They were filtered by p-value (correction by Benjamini, p-val < = 0.05) and by minimum number of genes belonging to each annotation term (count = 2).

To perform a comparative analysis of the disorder of the GO classes common to Arabidopsis and human, we quantified the “disorder” of a given GO class in each organism with the criterion described above. Then, a 2×2 contingency table was constructed containing the disordered proteins (*d)* for each of the two organisms (*A. th.* and *H. sa*.) and the complementary figures (number of “non-disordered” proteins according to that criteria, 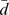) (Figure 6). We measured the significance of the difference between the observed and the expected frequencies of disordered proteins in *A. thaliana* and *H. sapiens* with a Pearson’s Chi-squared test with Yates’ continuity correction [54]. We consider only the classes for which the number of disordered proteins in Arabidopsis is higher (5% or more) than the “expected” value reported by the Chi-squared test, in order to take only the classes for which the difference in disorder is positive for Arabidopsis.

With this procedure, each GO functional class was assigned a p-value, which was corrected using the Benjamini & Hochberg multiple testing correction [55]. GO classes with low p-values correspond to those significantly enriched in disordered proteins in Arabidopsis when compared with human.

For these GO classes, we additionally calculated the average disorder of the orthologous proteins in both organisms. This was done in order to verify that the eventual differences in disorder are maintained when considering only the “comparable” proteins, and discard that these differences might be due to biases in the GO annotations of these two genomes. Orthology relationships between A. thaliana and H. sapiens proteins were taken from the InParanoid database v.7 [56]. InParanoid provides a collection of intra-organism paralog groups for most sequenced eukaryotic genomes, related by inter-organism orthology relationships. The differences in disorder content for these subsets of orthologous proteins were statistically assessed with a two-tailed t-test (to check the null hypothesis that there is no difference between the average disorder values) and a one-tailed t-test (null hypothesis that the disorder of the Arabidopsis orthologs is not higher than that of Human).

A given GO term can show up in the first test (disordered in Arabidopsis) but not in the second one (comparison with human) if, for example, the “amount” of disorder is similar in both organisms. Conversely, a term could appear in the second test but not in the first one, meaning that, while the disorder content of that functional class is not especially high in Arabidopsis, it is still significantly higher than in human. The third possibility is a term showing up in both tests: this would be a class which is significantly enriched in disorder in Arabidopsis and that it is also more disordered than in human. The complete set of GO terms in each of these three categories is shown in Table 1S (Additional Data File S1).

In both cases, the set of significant GO terms reported by each test was used as input for the ReviGO tool [57] in order to reduce the number of terms to a smaller meaningful set. This computational approach “collapses” a set of GO terms based on several measures of semantic similarity by removing functional redundancies. The result is a smaller number of representative terms, easier to handle and interpret. These resulting terms correspond to the cluster representatives (each represented as a single rectangle), and their choice is unaffected by whether the terms are more general or more specific. The size of each rectangle (cluster representative) represents the “uniqueness” of the term. This measure assesses whether the term is an outlier when compared semantically to the whole list, that is, the frequency of the GO term in the underlying GO database. The clusters representatives are automatically joined into “superclusters” of loosely related terms visualized with different colors. Each supercluster is given a broader name that represents a generic function common to all clusters. This representation allows a multidimensional visualization of the terms, while discarding any overrepresentation of similar functional terms.

All statistical analyses to estimate significance were implemented in the statistical analysis programming language *R* (www.r-project.org). All data processing performed in this study was done with *ad-hoc* scripts written in the *Perl* programming language. All datasets are available upon request.

## Supporting Information

Additional Data File S1 **Contains supplementary figures and tables.**
(PDF)

Additional Data File S2 **A table listing the GO:BP terms enriched in disorder in A. thaliana.**
(HTML)

Additional Data File S3 **A table listing the GO:BP terms enriched in disorder in A. thaliana with respect to Human, with the corresponding number of disordered proteins and total number of proteins for each organism.**
(HTML)

Additional Data File S4 **A table containing the average disorder (according with 4 different metrics) for the orthologous proteins belonging to the GO classes found to be enriched in disorder in A. thaliana respect to Human.**
(XLS)
