# Supplementary material for: Genome-Wide Analysis of Protein Disorder in Arabidopsis thaliana: Implications for Plant Environmental Adaptation
Source: PLoS One. 2013 Feb 7;8(2):e55524. doi: 10.1371/journal.pone.0055524 (PMC3567104; doi:10.1371/journal.pone.0055524)
Supplement: Additional Data File S1 — Contains supplementary figures and tables. (PDF) [file pone.0055524.s001.pdf]

## SUPPLEMENTAL MATERIAL

# Genome-wide analysis of protein disorder in *Arabidopsis thaliana*. Implications for environmental adaptation

Natalia Pietrosemoli[1,3], Juan A. García-Martín[2], Roberto Solano[2*] and Florencio Pazos[1*]

[1]Computational System Biology Group, National Centre for Biotechnology (CNB-CSIC).
[2] Plant Molecular Genetics Department, National Centre for Biotechnology (CNB-CSIC).
[3] Department of Bioengineering, Rice University. Houston, Texas.

Corresponding authors: fpazos@cnb.csic.es, rsolano@cnb.csic.es

| | **Mean content of disorder (%)** | | **Mean number of LDW** | | **Proteins with at least one LDW (%)** | | **Mean number of residues belonging to LDWs** | |
|---|---|---|---|---|---|---|---|---|
| | *A.th.* | *H.sa.* | *A.th.* | *H.sa.* | *A.th.* | *H.sa.* | *A.th.* | *H.sa.* |
| Iupred (short) | 17.0 | 22.4 | 0.6 | 1.4 | 36.3 | 60.9 | 9.0 | 14.9 |
| Iupred (long) | 16.8 | 24.5 | 0.6 | 1.3 | 32.8 | 56.6 | 10.5 | 17.6 |
| VSL2 | 38.9 | 44.9 | 1.2 | 1.9 | 68.1 | 78.9 | 26.1 | 34.4 |

**Table 1S. Summary of intrinsic disorder metrics for *A. thaliana and H. sapiens*.** Results shown for the different prediction methods.

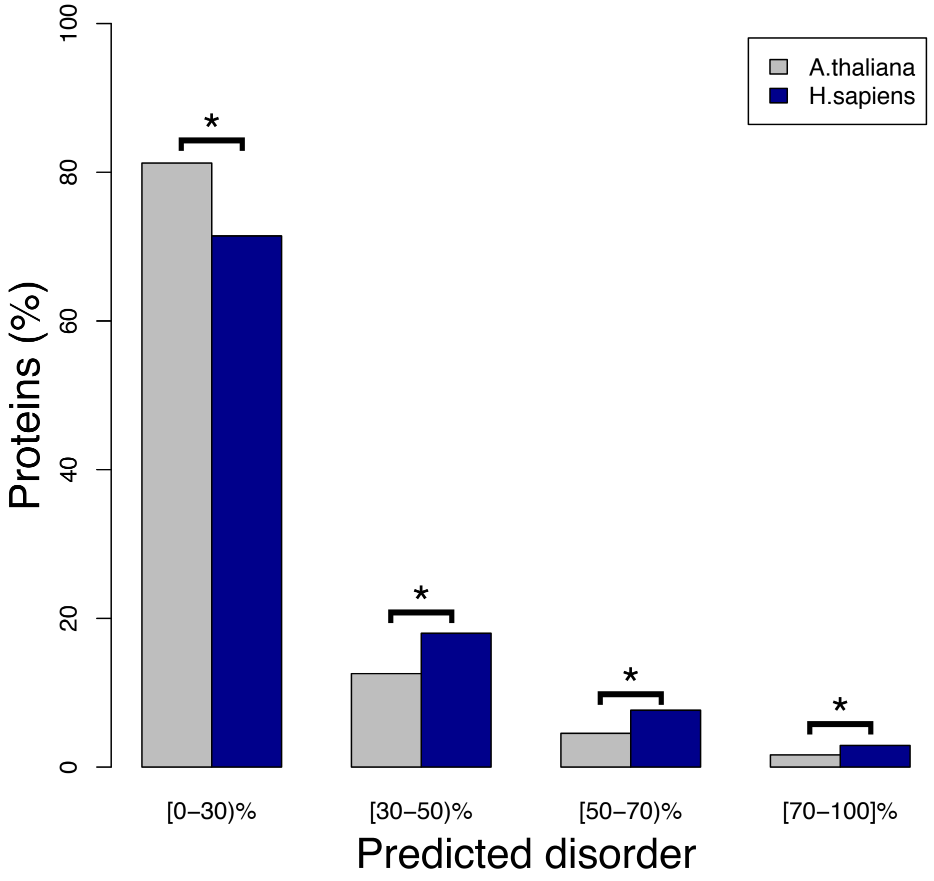


**Figure 1S. Fraction of proteins with different degrees of predicted disorder in *A. thaliana* and Human.** Protein disorder (as the percentage of disordered residues with respect to the sequence length) is binned into different ranges. Data based on Iupred (short) predictions.

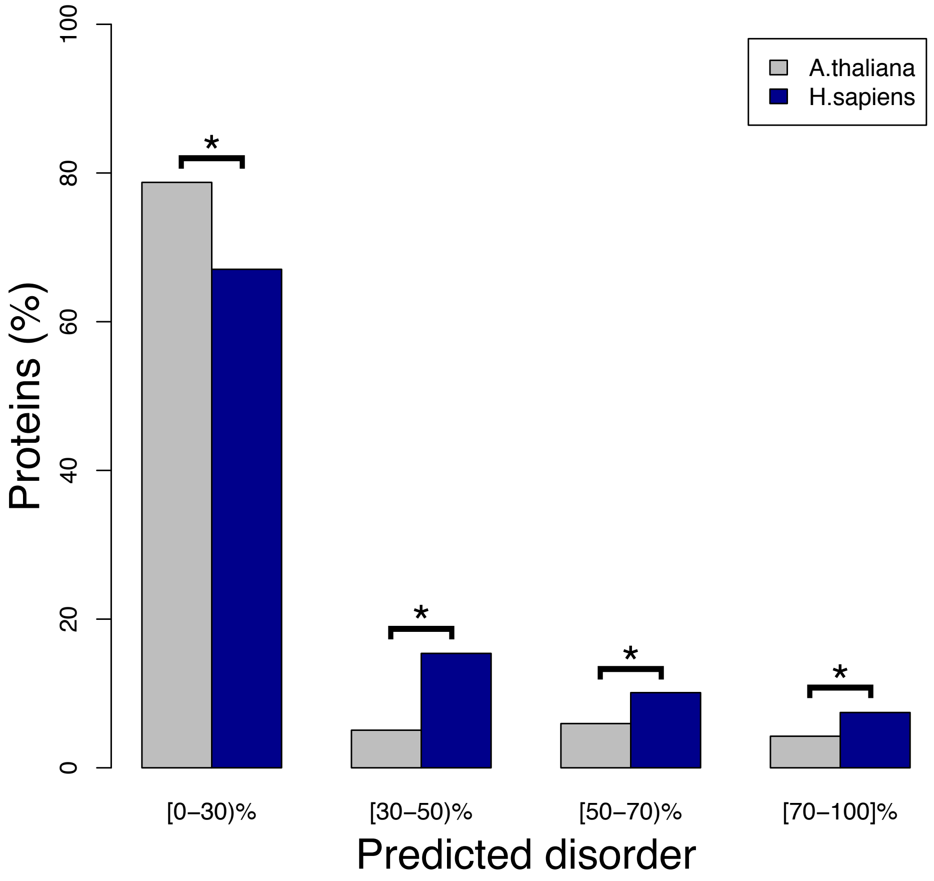


**Figure 2S. Fraction of proteins with different degrees of predicted disorder in *A. thaliana* and Human.** Protein disorder (as the percentage of disordered residues with respect to the sequence length) is binned into different ranges. Data based on Iupred (long) predictions.

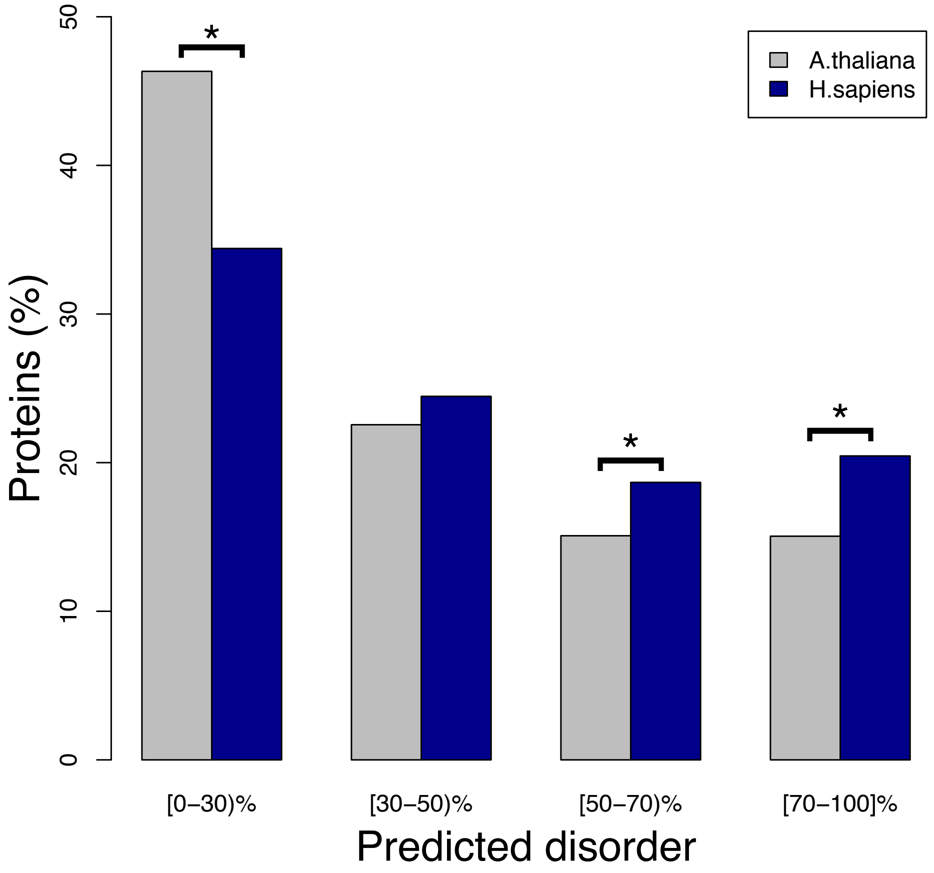


**Figure 3S. Fraction of proteins with different degrees of predicted disorder in *A. thaliana* and Human.** Protein disorder (as the percentage of disordered residues with respect to the sequence length) is binned into different ranges. Data based on VSL2 predictions.

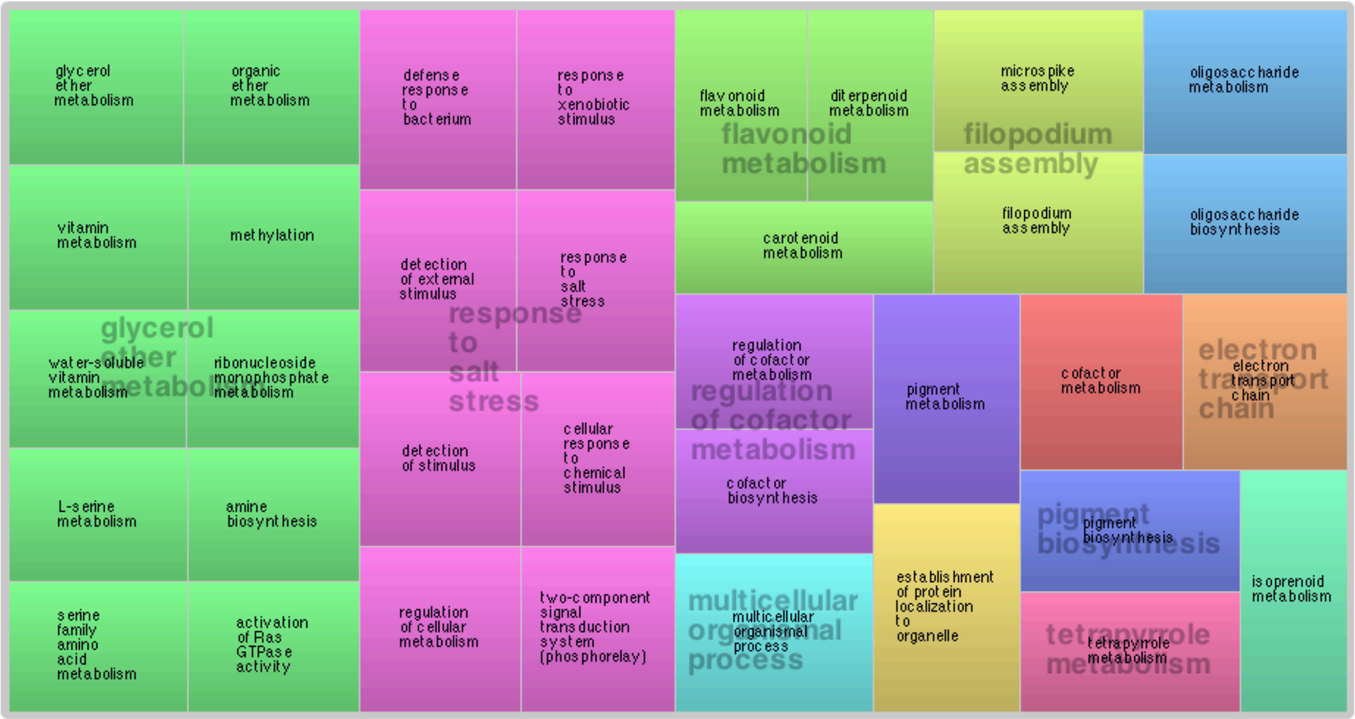


**Figure 4S. Representation of the GO "Biological Processes" comparatively enriched in disordered proteins in *A. thaliana* respect to *H. sapiens.*** Disordered proteins here are again those with 1 or more LDWs based on VSL2 predictions. Figure adapted from REVIGO, a system for summarizing and visualizing lists of GO terms. Each rectangle represents a cluster of related terms labelled according to a representative term. Rectangles are grouped in "superclusters" (identified with the same color) based on SimRel semantic similarity measure.

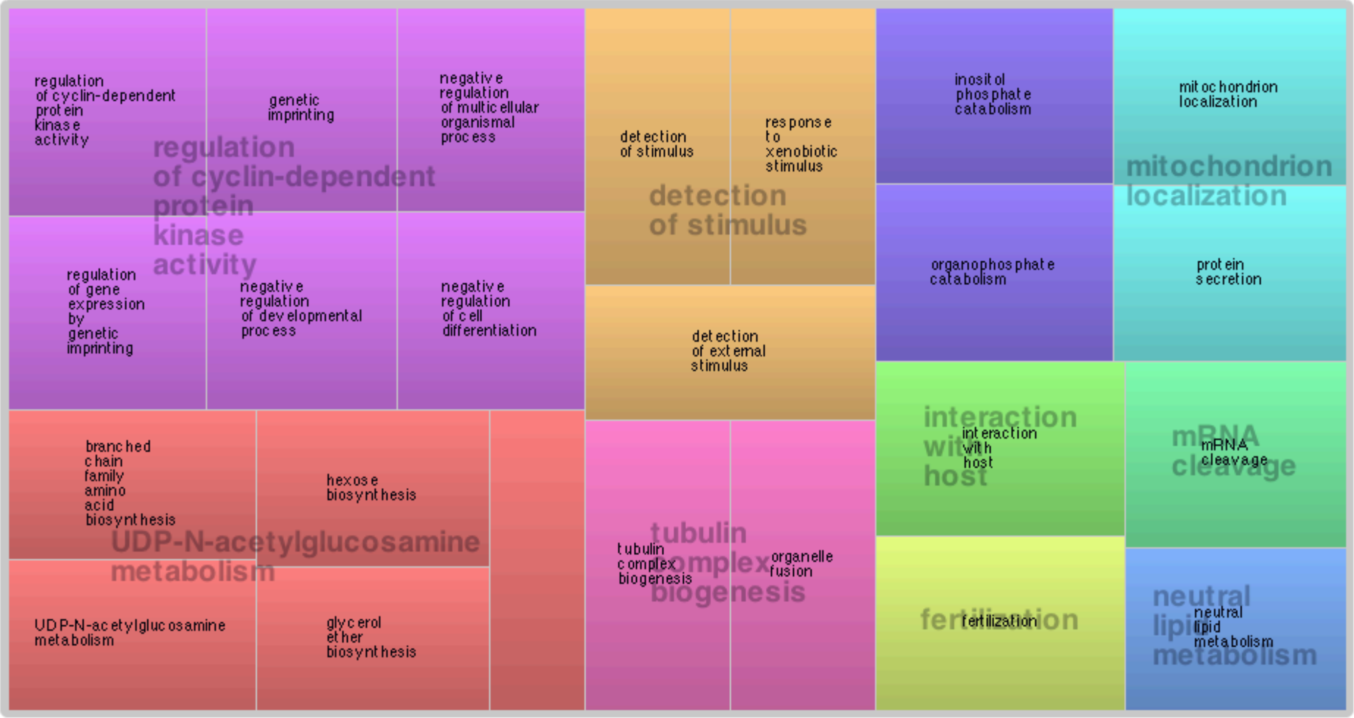


**Figure 5S. Representation of the GO "Biological Processes" comparatively enriched in disordered proteins in *A. thaliana* respect to *H. sapiens*.** Disordered proteins here are again those with 1 or more LDWs based on Iupred (option "long") predictions. Same REVIGO representation adaptation as in Figure 4S.

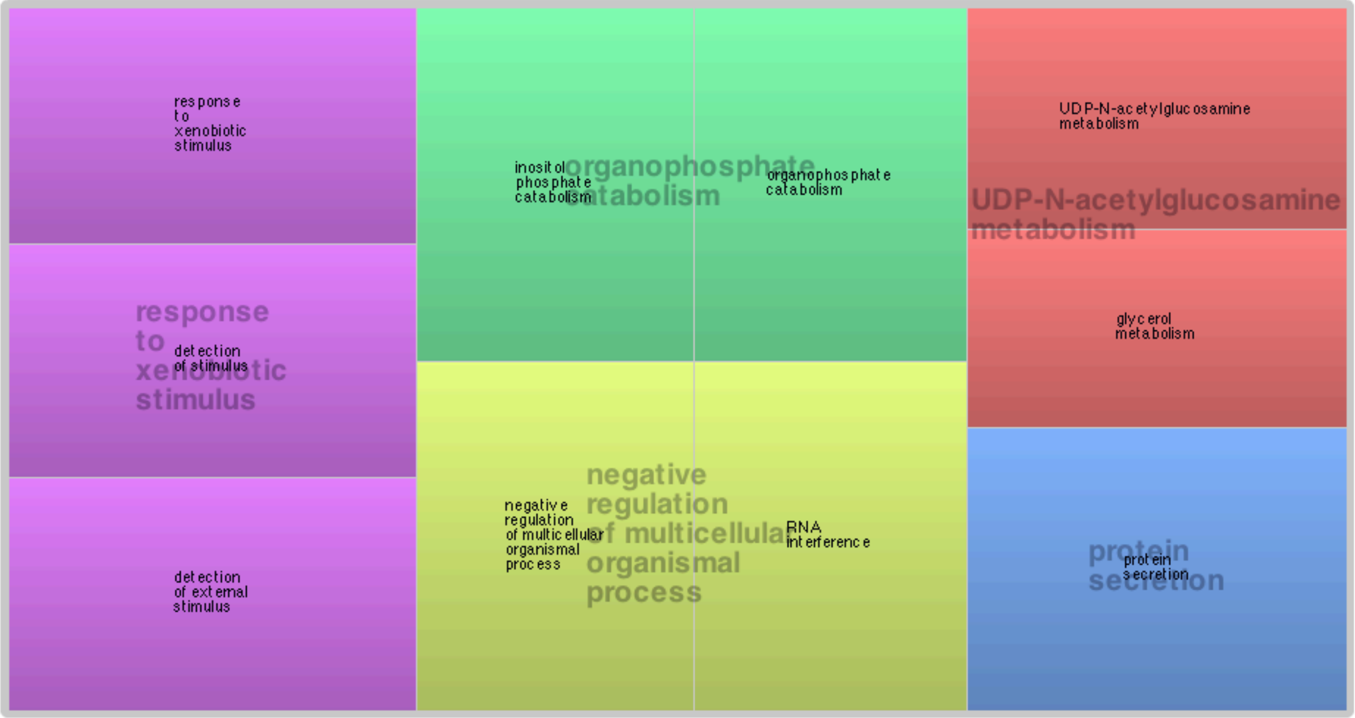

**Figure 6S. Representation of the GO “Biological Processes” comparatively enriched in disordered proteins in *A. thaliana* respect to *H. sapiens.*** Disordered proteins here are again those with 1 or more LDWs based on Iupred (option “short”) predictions.  Same REVIGO representation adaptation as in Figure 4S.

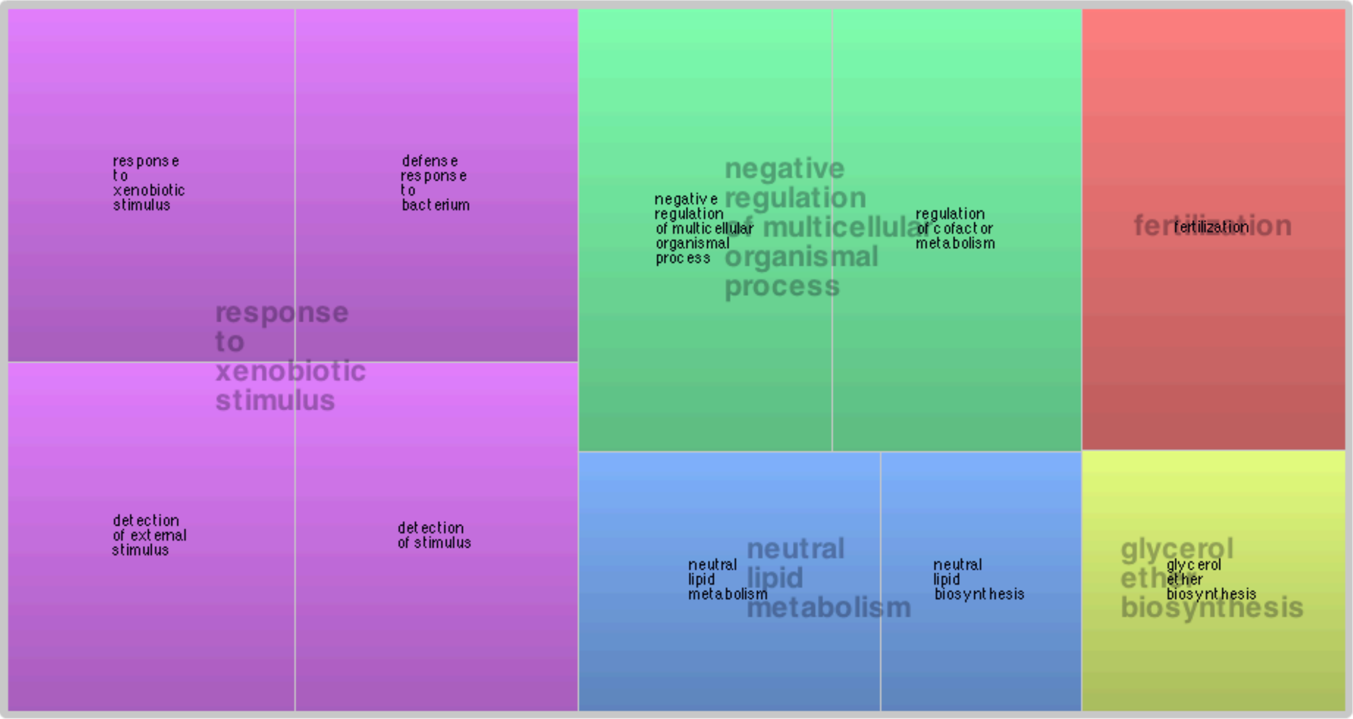


**Figure 7S.  Representation of the GO “Biological Processes” comparatively enriched in residues belonging to disordered binding regions (DBR) in *A. thaliana* with respect to *Human*.** DBR are calculated based on ANCHOR predictions. Same REVIGO representation adaptation as in Figure 4S.
