# Supplementary material for: Genome-Wide Analysis of Protein Disorder in Arabidopsis thaliana: Implications for Plant Environmental Adaptation
Source: PLoS One. 2013 Feb 7;8(2):e55524. doi: 10.1371/journal.pone.0055524 (PMC3567104; doi:10.1371/journal.pone.0055524)
Supplement: Additional Data File S2 — A table listing the GO:BP terms enriched in disorder in A. thaliana. (HTML) [file pone.0055524.s002.html]

REViGO Output


|  |  |  |  |  |  |
| --- | --- | --- | --- | --- | --- |
| term ID | description | frequency | uniqueness | dispensability | p-value |
| GO:0000003 | reproduction | 2.306 % | 1.00 | 0.00 | 9.33E-12 |
| GO:0007389 | pattern specification process | 0.066 % | 0.78 | 0.00 | 3.12E-06 |
| GO:0007623 | circadian rhythm | 0.016 % | 0.99 | 0.00 | 3.32E-05 |
| GO:0009058 | biosynthetic process | 31.173 % | 0.97 | 0.00 | 1.85E-06 |
| GO:0009639 | response to red or far red light | 0.014 % | 0.86 | 0.00 | 1.72E-05 |
| GO:0009266 | response to temperature stimulus | 0.144 % | 0.84 | 0.71 | 9.36E-05 |
| GO:0009314 | response to radiation | 0.185 % | 0.84 | 0.87 | 3.78E-11 |
| GO:0009416 | response to light stimulus | 0.173 % | 0.84 | 0.85 | 1.42E-09 |
| GO:0009987 | cellular process | 67.998 % | 1.00 | 0.00 | 8.84E-44 |
| GO:0016043 | cellular component organization | 3.677 % | 0.90 | 0.00 | 4.42E-06 |
| GO:0006323 | DNA packaging | 0.318 % | 0.79 | 0.73 | 1.42E-05 |
| GO:0034728 | nucleosome organization | 0.190 % | 0.87 | 0.92 | 3.74E-05 |
| GO:0016568 | chromatin modification | 0.112 % | 0.87 | 0.86 | 7.69E-08 |
| GO:0051276 | chromosome organization | 0.432 % | 0.87 | 0.75 | 7.27E-16 |
| GO:0006333 | chromatin assembly or disassembly | 0.195 % | 0.87 | 0.90 | 4.81E-05 |
| GO:0006325 | chromatin organization | 0.305 % | 0.87 | 0.84 | 1.22E-12 |
| GO:0006996 | organelle organization | 0.842 % | 0.87 | 0.71 | 9.14E-18 |
| GO:0016192 | vesicle-mediated transport | 0.348 % | 0.96 | 0.00 | 1.68E-05 |
| GO:0022406 | membrane docking | 0.029 % | 0.97 | 0.00 | 3.81E-07 |
| GO:0022414 | reproductive process | 2.253 % | 0.94 | 0.00 | 2.48E-13 |
| GO:0003006 | developmental process involved in reproduction | 0.141 % | 0.79 | 0.71 | 1.03E-17 |
| GO:0030005 | cellular di-, tri-valent inorganic cation homeostasis | 0.154 % | 0.99 | 0.00 | 7.47E-05 |
| GO:0032501 | multicellular organismal process | 1.467 % | 0.99 | 0.00 | 1.76E-40 |
| GO:0032502 | developmental process | 1.847 % | 0.99 | 0.00 | 7.61E-33 |
| GO:0051179 | localization | 19.119 % | 0.99 | 0.00 | 1.27E-11 |
| GO:0055080 | cation homeostasis | 0.263 % | 0.83 | 0.00 | 7.88E-05 |
| GO:0065007 | biological regulation | 15.106 % | 0.99 | 0.00 | 1.25E-166 |
| GO:0043687 | post-translational protein modification | 0.013 % | 0.91 | 0.01 | 7.13E-38 |
| GO:0022402 | cell cycle process | 0.220 % | 0.96 | 0.02 | 9.05E-07 |
| GO:0051301 | cell division | 1.073 % | 0.96 | 0.02 | 7.98E-10 |
| GO:0007049 | cell cycle | 1.209 % | 0.96 | 0.03 | 5.54E-12 |
| GO:0043170 | macromolecule metabolic process | 35.190 % | 0.97 | 0.05 | 5.53E-51 |
| GO:0006807 | nitrogen compound metabolic process | 36.484 % | 0.97 | 0.05 | 1.63E-148 |
| GO:0044237 | cellular metabolic process | 56.715 % | 0.93 | 0.07 | 7.58E-30 |
| GO:0016310 | phosphorylation | 6.139 % | 0.91 | 0.07 | 2.30E-23 |
| GO:0006796 | phosphate-containing compound metabolic process | 6.733 % | 0.91 | 0.92 | 8.54E-28 |
| GO:0006793 | phosphorus metabolic process | 6.762 % | 0.93 | 0.09 | 6.62E-28 |
| GO:0009250 | glucan biosynthetic process | 0.149 % | 0.89 | 0.11 | 9.76E-05 |
| GO:0008380 | RNA splicing | 0.170 % | 0.87 | 0.13 | 3.80E-07 |
| GO:0043412 | macromolecule modification | 5.666 % | 0.90 | 0.15 | 6.08E-20 |
| GO:0006139 | nucleobase-containing compound metabolic process | 29.194 % | 0.87 | 0.18 | 3.73E-180 |
| GO:0044249 | cellular biosynthetic process | 29.566 % | 0.87 | 0.21 | 7.36E-11 |
| GO:0032940 | secretion by cell | 0.614 % | 0.92 | 0.25 | 2.13E-06 |
| GO:0048278 | vesicle docking | 0.028 % | 0.93 | 0.99 | 3.81E-07 |
| GO:0006887 | exocytosis | 0.051 % | 0.92 | 0.80 | 6.48E-08 |
| GO:0006904 | vesicle docking involved in exocytosis | 0.024 % | 0.92 | 0.75 | 6.85E-07 |
| GO:0010646 | regulation of cell communication | 0.175 % | 0.80 | 0.26 | 5.08E-05 |
| GO:0009719 | response to endogenous stimulus | 0.216 % | 0.86 | 0.26 | 4.97E-32 |
| GO:0046903 | secretion | 0.626 % | 0.96 | 0.27 | 2.13E-06 |
| GO:0048518 | positive regulation of biological process | 0.523 % | 0.81 | 0.29 | 2.57E-06 |
| GO:0030001 | metal ion transport | 1.425 % | 0.95 | 0.29 | 8.72E-08 |
| GO:0051173 | positive regulation of nitrogen compound metabolic process | 0.275 % | 0.70 | 0.29 | 6.84E-07 |
| GO:0048522 | positive regulation of cellular process | 0.466 % | 0.72 | 0.94 | 2.90E-06 |
| GO:0031328 | positive regulation of cellular biosynthetic process | 0.289 % | 0.69 | 0.97 | 1.69E-05 |
| GO:0031325 | positive regulation of cellular metabolic process | 0.325 % | 0.70 | 0.98 | 3.09E-07 |
| GO:0045935 | positive regulation of nucleobase-containing compound metabolic process | 0.272 % | 0.68 | 0.97 | 6.84E-07 |
| GO:0010628 | positive regulation of gene expression | 0.214 % | 0.69 | 0.94 | 9.03E-07 |
| GO:0045893 | positive regulation of transcription, DNA-dependent | 0.204 % | 0.66 | 0.97 | 1.47E-06 |
| GO:0009893 | positive regulation of metabolic process | 0.337 % | 0.72 | 0.90 | 1.94E-07 |
| GO:0010604 | positive regulation of macromolecule metabolic process | 0.317 % | 0.69 | 0.99 | 7.48E-07 |
| GO:0009891 | positive regulation of biosynthetic process | 0.290 % | 0.71 | 0.97 | 1.69E-05 |
| GO:0010557 | positive regulation of macromolecule biosynthetic process | 0.225 % | 0.69 | 0.98 | 1.17E-05 |
| GO:0010467 | gene expression | 17.649 % | 0.88 | 0.29 | 4.93E-22 |
| GO:0016071 | mRNA metabolic process | 0.719 % | 0.86 | 0.30 | 9.96E-08 |
| GO:0006457 | protein folding | 0.973 % | 0.88 | 0.30 | 1.59E-13 |
| GO:0009628 | response to abiotic stimulus | 0.404 % | 0.85 | 0.34 | 7.13E-16 |
| GO:0006259 | DNA metabolic process | 7.219 % | 0.83 | 0.35 | 2.36E-11 |
| GO:0010033 | response to organic substance | 0.355 % | 0.81 | 0.35 | 8.39E-38 |
| GO:0042221 | response to chemical stimulus | 1.876 % | 0.83 | 0.42 | 1.80E-14 |
| GO:0006810 | transport | 18.616 % | 0.94 | 0.46 | 3.28E-10 |
| GO:0051234 | establishment of localization | 18.625 % | 0.94 | 0.80 | 1.04E-10 |
| GO:0044260 | cellular macromolecule metabolic process | 30.865 % | 0.84 | 0.47 | 1.14E-71 |
| GO:0010200 | response to chitin | 0.003 % | 0.83 | 0.49 | 3.80E-10 |
| GO:0006974 | response to DNA damage stimulus | 1.944 % | 0.79 | 0.50 | 3.27E-11 |
| GO:0006284 | base-excision repair | 0.230 % | 0.75 | 0.75 | 1.16E-05 |
| GO:0006281 | DNA repair | 1.921 % | 0.69 | 0.95 | 3.02E-11 |
| GO:0080090 | regulation of primary metabolic process | 9.227 % | 0.68 | 0.51 | 0.00E+00 |
| GO:0010468 | regulation of gene expression | 8.982 % | 0.64 | 0.88 | 6.24E-292 |
| GO:0031323 | regulation of cellular metabolic process | 9.216 % | 0.67 | 0.88 | 0 |
| GO:0031326 | regulation of cellular biosynthetic process | 8.772 % | 0.65 | 0.87 | 0 |
| GO:0051252 | regulation of RNA metabolic process | 8.553 % | 0.60 | 0.89 | 7.57E-177 |
| GO:0019219 | regulation of nucleobase-containing compound metabolic process | 8.854 % | 0.64 | 0.90 | 0 |
| GO:0006355 | regulation of transcription, DNA-dependent | 8.530 % | 0.59 | 0.91 | 4.50E-178 |
| GO:0060255 | regulation of macromolecule metabolic process | 9.279 % | 0.64 | 0.88 | 8.94E-282 |
| GO:0009889 | regulation of biosynthetic process | 8.776 % | 0.68 | 0.87 | 0 |
| GO:0010556 | regulation of macromolecule biosynthetic process | 8.751 % | 0.63 | 0.91 | 0 |
| GO:0051171 | regulation of nitrogen compound metabolic process | 8.859 % | 0.67 | 0.87 | 0 |
| GO:0009966 | regulation of signal transduction | 0.439 % | 0.68 | 0.51 | 1.03E-05 |
| GO:0051056 | regulation of small GTPase mediated signal transduction | 0.256 % | 0.69 | 0.94 | 4.50E-05 |
| GO:0046578 | regulation of Ras protein signal transduction | 0.214 % | 0.70 | 0.92 | 4.50E-05 |
| GO:0006464 | protein modification process | 3.999 % | 0.85 | 0.52 | 4.85E-29 |
| GO:0006468 | protein phosphorylation | 2.186 % | 0.84 | 0.77 | 1.09E-29 |
| GO:0016070 | RNA metabolic process | 13.956 % | 0.81 | 0.53 | 2.61E-06 |
| GO:0016567 | protein ubiquitination | 0.108 % | 0.89 | 0.54 | 1.55E-05 |
| GO:0034645 | cellular macromolecule biosynthetic process | 19.248 % | 0.80 | 0.56 | 1.49E-22 |
| GO:0009059 | macromolecule biosynthetic process | 19.469 % | 0.85 | 0.56 | 1.11E-22 |
| GO:0034641 | cellular nitrogen compound metabolic process | 35.144 % | 0.87 | 0.56 | 5.70E-162 |
| GO:0009751 | response to salicylic acid stimulus | 0.007 % | 0.82 | 0.57 | 8.01E-06 |
| GO:0048580 | regulation of post-embryonic development | 0.014 % | 0.68 | 0.58 | 2.40E-05 |
| GO:0009739 | response to gibberellin stimulus | 0.005 % | 0.82 | 0.58 | 8.21E-06 |
| GO:0048367 | shoot development | 0.016 % | 0.77 | 0.58 | 1.48E-09 |
| GO:0048827 | phyllome development | 0.011 % | 0.78 | 0.98 | 1.47E-10 |
| GO:0048366 | leaf development | 0.010 % | 0.78 | 0.97 | 6.54E-10 |
| GO:0009887 | organ morphogenesis | 0.103 % | 0.76 | 0.72 | 2.05E-10 |
| GO:0009965 | leaf morphogenesis | 0.004 % | 0.79 | 0.93 | 6.66E-06 |
| GO:0010016 | shoot morphogenesis | 0.007 % | 0.78 | 0.95 | 1.45E-06 |
| GO:0016044 | cellular membrane organization | 0.166 % | 0.90 | 0.59 | 5.17E-06 |
| GO:0022621 | shoot system development | 0.016 % | 0.79 | 0.59 | 1.29E-09 |
| GO:0065004 | protein-DNA complex assembly | 0.191 % | 0.88 | 0.60 | 3.48E-05 |
| GO:0006334 | nucleosome assembly | 0.188 % | 0.78 | 1.00 | 3.74E-05 |
| GO:0030154 | cell differentiation | 0.376 % | 0.76 | 0.61 | 2.35E-16 |
| GO:0048513 | organ development | 0.341 % | 0.75 | 0.86 | 2.63E-16 |
| GO:0048856 | anatomical structure development | 1.477 % | 0.76 | 0.80 | 7.21E-21 |
| GO:0048869 | cellular developmental process | 1.125 % | 0.75 | 0.90 | 1.09E-07 |
| GO:0050793 | regulation of developmental process | 0.854 % | 0.61 | 0.87 | 2.67E-10 |
| GO:0007275 | multicellular organismal development | 0.897 % | 0.76 | 0.88 | 2.40E-43 |
| GO:0048468 | cell development | 0.175 % | 0.77 | 0.74 | 3.63E-06 |
| GO:0009653 | anatomical structure morphogenesis | 1.066 % | 0.76 | 0.89 | 5.67E-06 |
| GO:0048731 | system development | 0.547 % | 0.75 | 0.83 | 2.05E-16 |
| GO:0051239 | regulation of multicellular organismal process | 0.211 % | 0.70 | 0.62 | 4.28E-07 |
| GO:0000160 | two-component signal transduction system (phosphorelay) | 2.351 % | 0.64 | 0.62 | 7.46E-26 |
| GO:0035556 | intracellular signal transduction | 2.601 % | 0.63 | 0.74 | 4.18E-19 |
| GO:0007165 | signal transduction | 5.494 % | 0.60 | 0.83 | 1.01E-07 |
| GO:0009873 | ethylene mediated signaling pathway | 0.004 % | 0.71 | 0.62 | 1.06E-27 |
| GO:0006397 | mRNA processing | 0.620 % | 0.85 | 0.62 | 1.69E-05 |
| GO:0051716 | cellular response to stimulus | 7.636 % | 0.79 | 0.63 | 1.01E-20 |
| GO:0009743 | response to carbohydrate stimulus | 0.027 % | 0.81 | 0.63 | 1.61E-11 |
| GO:0009755 | hormone-mediated signaling pathway | 0.107 % | 0.65 | 0.96 | 1.90E-33 |
| GO:0009753 | response to jasmonic acid stimulus | 0.009 % | 0.81 | 0.79 | 1.78E-07 |
| GO:0009737 | response to abscisic acid stimulus | 0.026 % | 0.80 | 0.84 | 2.45E-07 |
| GO:0009734 | auxin mediated signaling pathway | 0.016 % | 0.69 | 0.82 | 8.97E-07 |
| GO:0009733 | response to auxin stimulus | 0.024 % | 0.80 | 0.84 | 9.50E-07 |
| GO:0009725 | response to hormone stimulus | 0.194 % | 0.78 | 0.78 | 9.42E-32 |
| GO:0009723 | response to ethylene stimulus | 0.010 % | 0.81 | 0.79 | 8.90E-35 |
| GO:0032870 | cellular response to hormone stimulus | 0.134 % | 0.76 | 0.95 | 1.90E-33 |
| GO:0009791 | post-embryonic development | 0.098 % | 0.78 | 0.66 | 9.52E-19 |
| GO:0006351 | transcription, DNA-dependent | 10.059 % | 0.77 | 0.67 | 7.45E-237 |
| GO:0048608 | reproductive structure development | 0.079 % | 0.78 | 0.67 | 1.04E-15 |
| GO:0009908 | flower development | 0.020 % | 0.77 | 0.84 | 5.05E-10 |
| GO:0048467 | gynoecium development | 0.004 % | 0.78 | 0.93 | 4.41E-08 |
| GO:0048440 | carpel development | 0.003 % | 0.78 | 0.85 | 1.65E-07 |
| GO:0048438 | floral whorl development | 0.007 % | 0.78 | 0.78 | 1.96E-05 |
| GO:0019222 | regulation of metabolic process | 9.780 % | 0.73 | 0.70 | 1.16E-283 |
| GO:0050794 | regulation of cellular process | 14.067 % | 0.70 | 0.76 | 3.14E-211 |
| GO:0050789 | regulation of biological process | 14.662 % | 0.73 | 0.83 | 4.64E-188 |
| GO:0009790 | embryo development | 0.162 % | 0.77 | 0.70 | 7.97E-05 |
