# Supplementary material for: Genome-Wide Analysis of Protein Disorder in Arabidopsis thaliana: Implications for Plant Environmental Adaptation
Source: PLoS One. 2013 Feb 7;8(2):e55524. doi: 10.1371/journal.pone.0055524 (PMC3567104; doi:10.1371/journal.pone.0055524)
Supplement: Additional Data File S3 — A table listing the GO:BP terms enriched in disorder in A. thaliana with respect to Human, with the corresponding number of disordered proteins and total number of proteins for each organism. (HTML) [file pone.0055524.s003.html]

| term ID | description | frequency | uniqueness | dispensability | p-value | Number disordered proteins Ath | Total number of proteins Ath | Number disordered proteins Hsa | Total number of proteins Hsa | | |
| GO:0006826 | iron ion transport | 0.19% | 0.96 | 0 | 8.96E-03 | 19 | 23 | 44 | 101 |  |  |
| GO:0009581 | detection of external stimulus | 0.11% | 0.93 | 0 | 4.84E-02 | 48 | 64 | 33 | 64 |  |  |
| GO:0019748 | secondary metabolic process | 0.08% | 0.96 | 0 | 2.25E-02 | 141 | 372 | 18 | 86 |  |  |
| GO:0055072 | iron ion homeostasis | 0.17% | 0.85 | 0 | 1.79E-02 | 18 | 20 | 56 | 108 |  |  |
| GO:0065007 | biological regulation | 15.11% | 0.98 | 0 | 1.11E-02 | 3356 | 4278 | 10043 | 13190 |  |  |
| GO:0009812 | flavonoid metabolic process | 0.01% | 0.93 | 0.01 | 1.74E-02 | 31 | 58 | 0 | 11 |  |  |
| GO:0046434 | organophosphate catabolic process | 0.02% | 0.95 | 0.01 | 3.20E-02 | 6 | 6 | 2 | 11 |  |  |
| GO:0042440 | pigment metabolic process | 0.32% | 0.96 | 0.01 | 1.76E-03 | 110 | 154 | 36 | 78 |  |  |
| GO:0071941 | nitrogen cycle metabolic process | 0.17% | 0.88 | 0.01 | 1.71E-02 | 25 | 46 | 1 | 15 |  |  |
| GO:0006662 | glycerol ether metabolic process | 0.18% | 0.83 | 0.01 | 2.62E-03 | 15 | 15 | 56 | 116 |  |  |
| GO:0006639 | acylglycerol metabolic process | 0.02% | 0.74 | 0.86 | 3.12E-02 | 10 | 10 | 54 | 109 |  |  |
| GO:0006641 | triglyceride metabolic process | 0.01% | 0.76 | 0.94 | 2.33E-02 | 10 | 10 | 49 | 103 |  |  |
| GO:0006807 | nitrogen compound metabolic process | 36.48% | 0.95 | 0.02 | 1.10E-02 | 3640 | 4623 | 6458 | 8458 |  |  |
| GO:0006081 | cellular aldehyde metabolic process | 0.12% | 0.92 | 0.03 | 1.58E-02 | 13 | 26 | 4 | 34 |  |  |
| GO:0006638 | neutral lipid metabolic process | 0.02% | 0.84 | 0.03 | 3.12E-02 | 10 | 10 | 54 | 109 |  |  |
| GO:0051186 | cofactor metabolic process | 3.54% | 0.89 | 0.05 | 1.06E-02 | 197 | 355 | 138 | 319 |  |  |
| GO:0006457 | protein folding | 0.97% | 0.83 | 0.06 | 1.83E-03 | 210 | 275 | 179 | 289 |  |  |
| GO:0022900 | electron transport chain | 4.73% | 0.88 | 0.08 | 2.84E-03 | 126 | 226 | 66 | 175 |  |  |
| GO:0042726 | flavin-containing compound metabolic process | 0.22% | 0.79 | 0.1 | 2.29E-02 | 8 | 11 | 0 | 9 |  |  |
| GO:0000377 | RNA splicing, via transesterification reactions with bulged adenosine as nucleophile | 0.04% | 0.8 | 0.13 | 4.06E-02 | 35 | 35 | 215 | 267 |  |  |
| GO:0000375 | RNA splicing, via transesterification reactions | 0.04% | 0.8 | 0.81 | 4.52E-02 | 35 | 35 | 221 | 273 |  |  |
| GO:0006139 | nucleobase-containing compound metabolic process | 29.19% | 0.74 | 0.18 | 1.96E-12 | 3275 | 3870 | 6163 | 7807 |  |  |
| GO:0010467 | gene expression | 17.65% | 0.86 | 0.21 | 4.37E-02 | 3017 | 3612 | 5277 | 6480 |  |  |
| GO:0018904 | organic ether metabolic process | 0.18% | 0.86 | 0.22 | 2.62E-03 | 15 | 15 | 56 | 116 |  |  |
| GO:0019400 | alditol metabolic process | 0.31% | 0.81 | 0.23 | 4.82E-02 | 20 | 29 | 16 | 45 |  |  |
| GO:0032940 | secretion by cell | 0.61% | 0.91 | 0.24 | 9.59E-03 | 52 | 56 | 593 | 812 |  |  |
| GO:0006887 | exocytosis | 0.05% | 0.92 | 0.8 | 2.70E-03 | 45 | 47 | 262 | 371 |  |  |
| GO:0009410 | response to xenobiotic stimulus | 0.02% | 0.92 | 0.26 | 6.28E-04 | 5 | 7 | 23 | 192 |  |  |
| GO:0046903 | secretion | 0.63% | 0.96 | 0.27 | 2.65E-02 | 52 | 57 | 694 | 941 |  |  |
| GO:0042364 | water-soluble vitamin biosynthetic process | 1.15% | 0.72 | 0.27 | 2.13E-03 | 33 | 72 | 2 | 30 |  |  |
| GO:0006520 | cellular amino acid metabolic process | 5.56% | 0.64 | 0.89 | 4.56E-02 | 253 | 496 | 214 | 502 |  |  |
| GO:0008652 | cellular amino acid biosynthetic process | 3.17% | 0.58 | 0.93 | 2.05E-06 | 135 | 244 | 30 | 116 |  |  |
| GO:0016053 | organic acid biosynthetic process | 3.99% | 0.66 | 0.86 | 1.43E-02 | 264 | 510 | 124 | 305 |  |  |
| GO:0043436 | oxoacid metabolic process | 7.03% | 0.71 | 0.92 | 3.37E-02 | 455 | 914 | 422 | 971 |  |  |
| GO:0046394 | carboxylic acid biosynthetic process | 3.97% | 0.66 | 0.72 | 1.43E-02 | 264 | 510 | 124 | 305 |  |  |
| GO:0019752 | carboxylic acid metabolic process | 7.03% | 0.71 | 0.84 | 3.37E-02 | 455 | 914 | 422 | 971 |  |  |
| GO:0009067 | aspartate family amino acid biosynthetic process | 0.69% | 0.64 | 0.75 | 7.75E-03 | 23 | 53 | 0 | 19 |  |  |
| GO:0042742 | defense response to bacterium | 0.09% | 0.92 | 0.29 | 1.32E-06 | 142 | 227 | 48 | 141 |  |  |
| GO:0009617 | response to bacterium | 0.12% | 0.92 | 0.72 | 2.51E-03 | 159 | 269 | 84 | 199 |  |  |
| GO:0006183 | GTP biosynthetic process | 0.04% | 0.7 | 0.29 | 2.25E-02 | 5 | 7 | 1 | 17 |  |  |
| GO:0046131 | pyrimidine ribonucleoside metabolic process | 0.18% | 0.67 | 0.33 | 4.44E-02 | 9 | 12 | 6 | 25 |  |  |
| GO:0009220 | pyrimidine ribonucleotide biosynthetic process | 0.17% | 0.63 | 0.88 | 1.66E-02 | 9 | 12 | 5 | 26 |  |  |
| GO:0072528 | pyrimidine-containing compound biosynthetic process | 0.58% | 0.65 | 0.94 | 5.24E-03 | 21 | 28 | 17 | 51 |  |  |
| GO:0046051 | UTP metabolic process | 0.04% | 0.69 | 1 | 2.25E-02 | 5 | 7 | 1 | 17 |  |  |
| GO:0006220 | pyrimidine nucleotide metabolic process | 0.62% | 0.64 | 0.95 | 4.06E-02 | 20 | 28 | 21 | 55 |  |  |
| GO:0006228 | UTP biosynthetic process | 0.04% | 0.65 | 0.9 | 2.25E-02 | 5 | 7 | 1 | 17 |  |  |
| GO:0006221 | pyrimidine nucleotide biosynthetic process | 0.55% | 0.6 | 0.84 | 7.82E-04 | 20 | 27 | 7 | 33 |  |  |
| GO:0006778 | porphyrin-containing compound metabolic process | 0.70% | 0.74 | 0.37 | 1.31E-03 | 75 | 96 | 17 | 39 |  |  |
| GO:0006779 | porphyrin-containing compound biosynthetic process | 0.68% | 0.67 | 0.97 | 1.83E-03 | 62 | 72 | 15 | 30 |  |  |
| GO:0033014 | tetrapyrrole biosynthetic process | 0.76% | 0.67 | 0.98 | 5.50E-03 | 65 | 78 | 15 | 30 |  |  |
| GO:0042168 | heme metabolic process | 0.10% | 0.78 | 0.81 | 1.72E-03 | 20 | 21 | 14 | 33 |  |  |
| GO:0006082 | organic acid metabolic process | 7.12% | 0.75 | 0.39 | 1.99E-02 | 455 | 915 | 428 | 995 |  |  |
| GO:0009059 | macromolecule biosynthetic process | 19.47% | 0.77 | 0.4 | 1.11E-04 | 2912 | 3521 | 4957 | 6268 |  |  |
| GO:0033013 | tetrapyrrole metabolic process | 0.77% | 0.77 | 0.41 | 3.25E-03 | 76 | 100 | 17 | 39 |  |  |
| GO:0009309 | amine biosynthetic process | 3.26% | 0.69 | 0.42 | 3.35E-04 | 141 | 272 | 57 | 178 |  |  |
| GO:0006414 | translational elongation | 0.67% | 0.78 | 0.43 | 1.50E-03 | 24 | 31 | 39 | 103 |  |  |
| GO:0046471 | phosphatidylglycerol metabolic process | 0.00% | 0.84 | 0.46 | 2.67E-02 | 3 | 4 | 0 | 14 |  |  |
| GO:0006289 | nucleotide-excision repair | 0.23% | 0.75 | 0.46 | 4.85E-02 | 36 | 40 | 72 | 107 |  |  |
| GO:0000160 | two-component signal transduction system (phosphorelay) | 2.35% | 0.71 | 0.48 | 2.36E-04 | 172 | 198 | 0 | 4 |  |  |
| GO:0090304 | nucleic acid metabolic process | 21.07% | 0.73 | 0.49 | 3.86E-16 | 3057 | 3487 | 5532 | 6818 |  |  |
| GO:0034645 | cellular macromolecule biosynthetic process | 19.25% | 0.69 | 0.49 | 3.61E-04 | 2908 | 3513 | 4897 | 6168 |  |  |
| GO:0016070 | RNA metabolic process | 13.96% | 0.68 | 0.49 | 5.21E-17 | 2759 | 3086 | 5048 | 6115 |  |  |
| GO:0006720 | isoprenoid metabolic process | 0.40% | 0.81 | 0.53 | 5.49E-06 | 90 | 152 | 19 | 79 |  |  |
| GO:0019222 | regulation of metabolic process | 9.78% | 0.73 | 0.54 | 1.14E-16 | 2468 | 2810 | 5585 | 6931 |  |  |
| GO:0050794 | regulation of cellular process | 14.07% | 0.7 | 0.76 | 2.92E-07 | 2929 | 3540 | 9119 | 11622 |  |  |
| GO:0050789 | regulation of biological process | 14.66% | 0.74 | 0.83 | 7.25E-05 | 3148 | 3916 | 9475 | 12302 |  |  |
| GO:0070887 | cellular response to chemical stimulus | 0.37% | 0.88 | 0.55 | 1.11E-03 | 579 | 832 | 792 | 1287 |  |  |
| GO:0034641 | cellular nitrogen compound metabolic process | 35.14% | 0.75 | 0.56 | 8.14E-05 | 3596 | 4490 | 6405 | 8349 |  |  |
| GO:0006721 | terpenoid metabolic process | 0.23% | 0.79 | 0.64 | 3.72E-08 | 74 | 119 | 7 | 54 |  |  |
| GO:0016108 | tetraterpenoid metabolic process | 0.04% | 0.81 | 0.79 | 2.42E-02 | 34 | 42 | 1 | 6 |  |  |
| GO:0016116 | carotenoid metabolic process | 0.04% | 0.81 | 0.79 | 2.42E-02 | 34 | 42 | 1 | 6 |  |  |
| GO:0043288 | apocarotenoid metabolic process | 0.00% | 0.83 | 0.66 | 1.59E-02 | 15 | 24 | 0 | 10 |  |  |
| GO:0071310 | cellular response to organic substance | 0.23% | 0.88 | 0.67 | 2.36E-02 | 548 | 706 | 626 | 879 |  |  |
| GO:0006351 | transcription, DNA-dependent | 10.06% | 0.63 | 0.67 | 3.25E-27 | 2361 | 2536 | 4154 | 4941 |  |  |
| GO:0032774 | RNA biosynthetic process | 10.14% | 0.65 | 0.74 | 1.42E-30 | 2361 | 2538 | 4189 | 5029 |  |  |
| GO:0016101 | diterpenoid metabolic process | 0.01% | 0.83 | 0.69 | 2.32E-02 | 12 | 25 | 6 | 44 |  |  |
| GO:0080090 | regulation of primary metabolic process | 9.23% | 0.65 | 0.7 | 6.96E-21 | 2320 | 2592 | 5157 | 6351 |  |  |
| GO:0009889 | regulation of biosynthetic process | 8.78% | 0.62 | 0.87 | 7.90E-23 | 2187 | 2392 | 4194 | 5077 |  |  |
| GO:0010468 | regulation of gene expression | 8.98% | 0.63 | 0.88 | 6.43E-22 | 2229 | 2430 | 4132 | 4963 |  |  |
| GO:0031323 | regulation of cellular metabolic process | 9.22% | 0.64 | 0.88 | 4.86E-25 | 2322 | 2564 | 5227 | 6411 |  |  |
| GO:0031326 | regulation of cellular biosynthetic process | 8.77% | 0.57 | 0.91 | 4.11E-22 | 2184 | 2387 | 4179 | 5044 |  |  |
| GO:0051252 | regulation of RNA metabolic process | 8.55% | 0.52 | 0.89 | 5.91E-21 | 2107 | 2280 | 3823 | 4549 |  |  |
| GO:0010556 | regulation of macromolecule biosynthetic process | 8.75% | 0.58 | 0.91 | 1.67E-24 | 2164 | 2353 | 4034 | 4872 |  |  |
| GO:0019219 | regulation of nucleobase-containing compound metabolic process | 8.85% | 0.56 | 0.9 | 1.00E-19 | 2174 | 2364 | 4325 | 5147 |  |  |
| GO:2000112 | regulation of cellular macromolecule biosynthetic process | 8.75% | 0.54 | 0.89 | 1.02E-22 | 2164 | 2353 | 3983 | 4787 |  |  |
| GO:0006355 | regulation of transcription, DNA-dependent | 8.53% | 0.49 | 0.91 | 1.32E-22 | 2102 | 2269 | 3711 | 4424 |  |  |
| GO:0060255 | regulation of macromolecule metabolic process | 9.28% | 0.65 | 0.88 | 6.64E-24 | 2314 | 2579 | 4926 | 6104 |  |  |
| GO:0051171 | regulation of nitrogen compound metabolic process | 8.86% | 0.6 | 0.87 | 8.66E-22 | 2185 | 2380 | 4359 | 5229 |  |  |
